# If You Cut It Will They Ride? Longitudinal Examination of the Elasticity of Public Transport Ridership in the Post-Pandemic Era

**DOI:** 10.1177/03611981241240754

**Published:** 2024-04-13

**Authors:** Paul Redelmeier, Ahmed El-Geneidy

**Affiliations:** 1 McGill University, Canada

**Keywords:** public transportation, sustainability and resilience, transportation and society, community resources and impacts, public transit, equity in transportation

## Abstract

In the past two years, public transport ridership has declined because of COVID-19 pandemic health measures and new working from home policies. This decline in ridership has caused major financial stress on public transport agencies around the world. Several agencies have responded to this financial stress by reducing services. The extent to which these service cuts will affect transit ridership is unknown because of the changing operational environment in the post-pandemic world. Our study uses a longitudinal panel data from Montréal, Quebec, Canada, to explore the relationship between route-level ridership and operational factors over time. We find that public transport ridership demand at the route level is highly elastic when compared to trip frequency and has become more elastic after the COVID-19 pandemic. Our findings imply that agencies cutting service in the post-pandemic era run a much more significant risk of creating a “doom spiral,” where service reductions spur greater declines in ridership, forcing further reductions. Demand was found to be most elastic on more frequent routes, so agencies should prioritize maintaining services on their core routes in the post-pandemic era. This study can be of use to public transit planners and policymakers considering making service changes to attract more riders or trying to respond to post-COVID-19 financial stress.

The COVID-19 pandemic has profoundly affected the way people move around cities, and no mode has suffered more than public transit (*1*). Transit agencies across North America have reported major reductions in ridership in 2020 because of public health concerns and increases in telework (*2*). By mid-2023, ridership levels in the U.S.A. had only recovered to ~70% of pre-pandemic levels, while those in Canada had recovered to just over 75% (*3*, *4*). Most North American agencies reduced service levels in the pandemic’s early months, when COVID-19 restrictions were strictest (*5*). By mid-2021, many had returned to pre-pandemic service levels in the hopes of luring ridership back (*6*). The funding to achieve this began to run out before ridership returned, and in 2022 many agencies made service cuts in the hopes of reducing budget deficits caused by lower ridership (*7*). This has prompted fears of a transit “doom spiral” (*8*). In that scenario, a vicious cycle ensues in which transit agencies cut services, making transit less convenient, leading to declines in ridership, forcing further reductions.

To understand the risk of this doom spiral, we must understand the relationship between post-pandemic ridership and service frequency. If demand is very sensitive—or *elastic*—then the risk is high, because it would mean that if transit agencies cut services, they will suffer a large reduction in ridership. If demand is *inelastic*, then services could be reduced without greatly decreasing farebox revenue, making cuts more viable. The elasticity of transit demand has been extensively studied for pre-pandemic ridership, but to our knowledge no studies have estimated it for post-pandemic periods. Our study aims to fill this gap through a longitudinal analysis of bus ridership in Montréal, Quebec, Canada.

## Literature Review

### Determinants of Public Transport Ridership

Previous research on transit has identified major determinants of ridership. Micro-scale studies have investigated the likelihood of transit use at the individual level, including the impact of socio-demographic characteristics, personal preferences, and the built environment (*9*, *10*). This research has identified subgroups who are more likely to take transit, including recent immigrants, students, and the unemployed (*11*, *12*). Recent studies have identified areas that were more likely to see ridership declines during the pandemic, including those with more white, educated, and high-income individuals (*13*, *14*).

Macro-scale studies examine the impact of municipal, regional, or national phenomena on transit ridership. These phenomena are generally split into internal and external factors. Internal factors are those that are under the control of a transit agency, while external factors relate to wider economic and political forces that affect society at large. The literature has identified several important internal factors. Service levels have been found to have a positive, significant relationship with ridership (*15 [Bibr bibr16-03611981241240754]*–*17*), while fares have been found to have a negative relationship (*18*). Other factors relate to service quality, including reliability, comfort, and convenience (*19 [Bibr bibr20-03611981241240754] [Bibr bibr21-03611981241240754]*–*22*).

The literature has identified several external factors. Population and employment rate have been found to have a statistically significant, positive relationship with ridership (*23*). Land use variables, including population density and parking availability, have been identified as contributors to ridership (*19*, *24*). Since the pandemic, rates of telework have been found to have a negative relationship with transit ridership (*2*, *25*). Researchers have found mixed results when examining the impact of emerging technologies, such as ride-hailing and bicycle-sharing, on public transit ridership (*18*, *24*, *26*). Per capita rates of auto ownership have a persistently negative impact (*15*, *27*), while gas prices have only sometimes been found to be impactful (*28 [Bibr bibr29-03611981241240754]*–*30*).

### Level of Analysis

Research on ridership occurs at several levels, ranging from the stop (*11*, *31*, *32*), stop-segment (*33*), and route levels (*16*, *22*, *34*, *35*) to the system level (*15*, *18*, *29*). Our study uses panel data to investigate ridership at the route level. This approach has been previously used to estimate the impact of COVID-19 on transit demand in the short term (i.e., up to December 2020) (*35*). It was used to estimate the pre-COVID-19 relationship between frequency of bus services and ridership (*16*). Analyzing transit at the route level mirrors the behavior of transit agencies, which often analyze and adjust service at this scale (*16*), which can help maximize the relevance of our research to practice.

The reverse relationship between ridership and frequency is a known challenge in the public transit literature. Service frequency can influence ridership (by making transit services more or less attractive), but ridership can also influence service frequency (if agencies make service changes to adjust to changes in ridership). Studies have dealt with this challenge in different ways. One approach is to use two-stage least squares regression models, in which the first regression is used to predict transit supply and then this predicted transit supply variable is used to predict ridership (*36*). Others, including this paper, have mitigated this endogeneity through the use of longitudinal panel data (*16*, *37*). As described in the *Methodology* section, longitudinal panel models split the error term into a time-invariant component and a time-varying component. In our case, this means that each route is allowed to have its own error term that does not vary over time. Since the majority of the ridership’s influence on service frequency does not vary over time, it is expected that this endogeneity will mostly be contained within this time-invariant term. This limits the effect of endogeneity on our model’s estimates.

## Study Context

**Figure 1.**
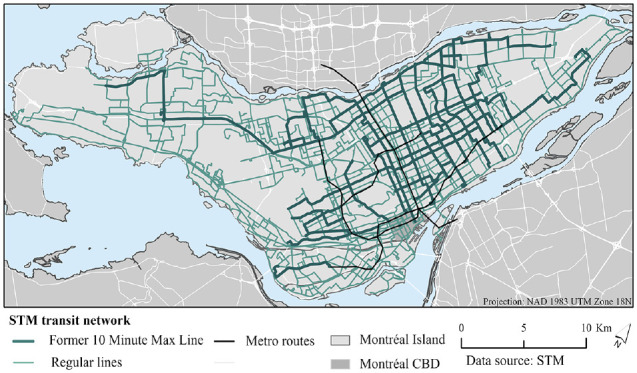
2022 Société de transport de Montréal (STM) system map. *Note*: CBD = central business district.

Montréal is Canada’s second-largest city, with 4.4 million people living in the greater Montréal area (*38*). Two million people live on the Island of Montréal, the densest part of the metropolitan region. Transit on the Island of Montréal is mostly provided by the Société de transport de Montréal (STM), which, as of December 2022, operated a network of 225 bus routes and four metro lines (*39*) (Figure 1). From 2010 to 2022, a subset of these lines was defined as 10-Minute Max, which made up a basic grid of frequent service. There are several other transit agencies in the region, including Exo, which operates a commuter rail network connecting the suburbs to the Island of Montréal.

Annual STM bus ridership decreased by 9.7% between 2010 and 2019 (*40*, *41*). This decline was not distributed equally consistently over time; for example, bus ridership in 2018 was 4% higher than in 2010, before declining by 13% between 2018 and 2019. To recover this decline in ridership, STM increased their service frequency by 0.7% between 2017 and 2019 (the period defined as pre-COVID-19 in our study). The ridership changes that occurred in the years before the pandemic occurred on routes that were different from the routes that experienced service cuts in the post-COVID-19 period.

**Figure 2.**
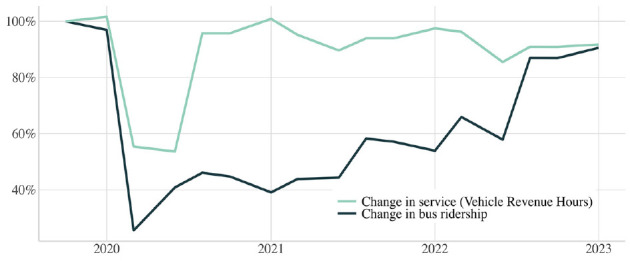
Change in average weekday bus ridership and service: October 2019–January 2023 (inclusive).

During COVID-19, bus ridership dropped to 25% of pre-pandemic levels in April 2020, before recovering to 90% as of January 2023 (Figure 2). After making cuts early in the pandemic, STM restored pre-pandemic service in 2021, stating: “for the first time in many years, supply is not being determined by demand, but by our moral obligation” (*42*). However, facing a $78 million CAD budget deficit, STM was forced to subsequently make “surgical” service cuts (*43*). A significant ridership recovery occurred in Fall 2022, the first non-summer period with no public health restrictions since the start of COVID-19.

## Data

### Ridership Data

Through an access to information request, ridership data were obtained from STM in the form of average weekday unlinked trips for each bus route and metro station. This study concentrates on changes in bus ridership only. This data span from January 2010 to January 2023 (inclusive). STM changes schedules five times a year—mid-January, mid-March, mid-June, mid-August, and late-October—to address changes in congestion levels, reliability issues in schedules, and seasonality. Bus data were provided at the route level for each of these periods.

Ridership data from January 2020 to March 2022 (inclusive) were excluded from the study sample because public health restrictions were in place during those periods (*44*). Thus, post-COVID-19 data were restricted to the four consecutive periods between June 2022 and January 2023 (inclusive). These data were then compared with ridership from pre-COVID-19 from June 2017 to January 2018 and from June 2018 to January 2019. As such, our study consists of all routes (233) in 12 periods, with the periods starting in June, August, October, and January each appearing three times in our dataset.

Certain routes were removed from the dataset before analysis. A total of 20 routes were cancelled or introduced between 2017 and 2023: these were removed. The study does not examine ridership changes on the system’s 23 night routes (300-series), which run between 2:00 and 5:00 a.m. These routes operate after the metro has closed, and have an entirely different spatial configuration than daytime routes. All 700+ series routes (13 routes) were excluded as they are designated as shuttle services, and mostly serve tourist destinations. Eight routes were removed for miscellaneous reasons: three did not run during the summer, two had no trips during the morning peak, one had no ridership data, and two ran along Boulevard Pie-IX, where a bus rapid transit (BRT) system opened midway during the study period. This reduced the dataset from 233 to 169 routes. A separate case was generated for our 12 periods, resulting in a sample of 2028 observations.

### Internal Variables

STM operation data for each of our 12 periods were retrieved from archived General Transit Feed Specification (GTFS) datasets available online (*45*). A different GTFS dataset representing each period’s schedule was retrieved for each date. The R package “tidytransit” was used to read the GTFS files into R (*46*). Scheduled travel time for each trip was calculated by subtracting the arrival time at the last stop from the departure time at the first stop; these were then averaged to calculate a route’s average scheduled travel time. As a route may run several different patterns throughout the day, mean trip distance was calculated for each route. This was done by finding the length in meters of each trip by using the “sf” package’s st_distance function (*47*), and then averaging these by route. Average trip speed was calculated by dividing each trip’s travel time (in hours) by its length (in kilometers). Each of these metrics were calculated for “peak trips”—defined as trips whose first stop departs between 6 and 9 a.m.

Several dummy variables were generated for each route. Two variables were created for express routes (400-series routes) and 10-Minute Max routes. The 10-Minute Max network consisted of 31 routes in 2017–2019, then shrank to 8 routes in 2022, before being scrapped all together in January 2023. The specific routes labeled as 10-Minute Max for each period were confirmed by reviewing archives of STM’s 10-Minute Max webpage via the Wayback Machine, which displays what a webpage looked like on a specific date. Routes were defined as intersecting with the metro and/or commuter rail systems if they stopped within a 200-m buffer of a metro or Exo station, respectively. Given that COVID-19-era trends in telecommuting have prominently affected central business districts (CBDs), a dummy variable was generated based on whether a bus route intersected with Montréal’s CBD. This was defined as the area east of Rue Guy, south of Rue Sherbrooke, west of Rue Saint-Denis, and north of the Saint-Lawrence river.

To control for the link between the transit system’s operations and land use, the study calculated accessibility at the route level for each period. In transport literature, accessibility refers to the ease of reaching destinations (*48*); individuals living in high accessibility areas can reach more activities in a limited time (*49*). Accessibility blends the transport system (e.g., the location, frequency, and speed of the transit network) with land use (e.g., the number and location of jobs). One of the most frequently used measures of accessibility is cumulative opportunities, which scores an area’s accessibility based on how many jobs can be reached *from* the area using a given mode within a predefined travel time threshold. This measure’s popularity is because of its ease of calculation, understandability by the public, and reliability against more complex measures (*50*, *51*). Although accessibility is typically generated at the system or regional level, we sought to evaluate accessibility at the route level, using a method inspired by Albuquerque-Oliveira et al. (*52*). We calculated, for each census tract (CT) within 400 m of a given route, the number of jobs that are accessible in 45 min using that same route (either directly, or with transfers to other routes). To aggregate these CT-level accessibility figures at the route level, we calculated a weighted average. This average was weighted based on the number of people from each CT that lived within 400 m of that route. The 45-min threshold was chosen as it is frequently used in transportation planning to measure regional accessibility (*53*). This was calculated at 20 times (i.e., at 8:00, 8:03, and until 8:57 a.m.), and then the median score was selected. This sampling method allowed us to calculate a more generalizable route accessibility than calculating accessibility at a single point in time. These estimates were calculated using the detailed_itineraries() function in r5r, an open-source package for generating multimodal transport routes in R (*54*).

### External Variables

Our study made use of demographic and socioeconomic data, sourced from Statistics Canada’s 2016 and 2021 censuses. These data points were sourced at the CT level for the entirety of the Island of Montréal. The following demographic variables were retrieved: population, population density, median household income ($), number of immigrants who had arrived in Canada in the previous five years, number of households paying more than 30% of their income on housing, unemployment rate (%), work from home (WFH) rate (%), and number of jobs. For all variables except for number of jobs and WFH rate, linear interpolation was used to generate the 2017–2019 variables, and linear extrapolation was used to generate the 2022–2023 variables. For number of jobs, the unadjusted 2016 figures were used for all periods. The 2021 job location data are unlikely to be accurate for 2022–2023, because rates of telework changed materially between 2021 and 2022, as COVID-19 restrictions were lifted (*55*). For WFH rate, the unadjusted 2016 value was used for 2017–2019, because there were no exogenous shocks that would indicate that 2016 figure would have meaningfully changed for those years. For 2022 and 2023, the unadjusted 2021 figure was used. As WFH rates in 2022 were lower than 2021, the 2022–2023 WFH rates represent the percentage of workers in a CT who have the *potential* to WFH (because they worked from home in 2021), rather than the percentage of workers who were *actually* working from home at that time.

The demographics of each route were estimated using an approach used by Diab et al. (*16*). A 400-m buffer was generated around each route and intersected with shapefiles of the 2016 CTs. This buffer was generated around the entire route, not just the route stops. This area represents the part of each CT that was in a given route’s catchment. This area was then divided by the total area of each CT to calculate the proportion of each CT in a given route’s catchment. The resulting ratio for each CT was then used to weight the demographic variables, so that they could be appropriately averaged or summed together for each route. This approach has one major drawback: it assumes CTs are homogenous. This is a limitation, as areas adjacent to bus routes are likely to be different than areas outside the bus routes’ 400-m buffer. However, it was deemed acceptable, as Montréal has not pursued a specific densification policy around its bus routes (in contrast to the city’s Transit-Oriented Development program around rapid transit stations).

To calculate the impact of bicycle-sharing on each route, the number of bicycle-sharing trips in each route’s catchment was calculated. BIXI, the company which operates bicycle-sharing in Montréal, provides the origin and date of each of its customers’ trips. For each period and route, all trips beginning at a station within a 400-m buffer of the route were counted. This was then divided by the number of days in that period during which BIXI was in operation to get the average number of bicycle-sharing trips per day for each route–period combination. In addition to these variables, average gas price and minimum wage were identified for each period in our sample. Three seasonal dummy variables were created, for June, August, and October, as was a dummy variable for whether COVID-19 had occurred yet. Table 1 shows summary statistics for the external and internal variables included in the final model specification, calculated in October of that year.

**Table 1.** Summary Statistics for Variables Included in Final Models

Variable name	Short form name	2017	2018	2022
Internal variables				
Daily ridership (total)	Ridership (total)	837,289	865,290	629,205
Daily ridership (mean)	Ridership (mean)	4954	5120	3723
Daily bus trips (mean)	Daily trips	100.97	100.72	90.35
Average travel time (min) (mean)	Travel time	36.40	36.59	37.38
Accessibility to jobs in 45 min (mean)	Accessibility	77,232	77,943	64,672
Route connects to metro (%) (dummy mean)	Connects to metro	79.29	79.88	81.66
Route connects to Exo (%) (dummy mean)	Connects to Exo	45.56	44.97	36.69
Route intersects CBD (%) (dummy mean)	Intersects CBD	13.61	13.61	13.61
Route is 10-Minute Max (%) (dummy mean)	10-Minute Max	17.75	17.75	4.73
External variables				
Median household income ($) CAD (mean)	Income	59,948	62,550	72,994
Recent immigrant population (mean)	Recent immigrants	3254	3140	2719
Unemployment rate (%) (mean)	Unemployment rate	9.25	9.51	10.62
Workers with potential to telework (%) (mean)	Work from home	9.73	9.73	41.58

*Note*: CBD = central business district.

## Methodology

All non-dummy variables, including the dependent variable ridership, were first transformed into their natural logarithmic form. This log–log formulation was done to enable analysis of model results with respect to elasticities, and not to improve model fit. For example, if an independent variable has a coefficient of 0.2, this implies that a 10% change in that independent variable would predict a 2% change in the dependent variable, all else being equal.

The data were then split into a training sample (75% of routes) and testing sample (25%). The model would be developed using the training sample, and then cross-validated on the testing sample to test the quality of the model’s predictions and ensure it was not overfitted. When splitting the full sample into the training sample and testing sample, a random stratified sampling process was followed. This ensured that the two samples had roughly equal percentages of routes that were classified as “connecting to Exo,”“Express,” and “10-Minute Max.”

The full set of independent variables was then pared down by eliminating highly colinear variables. The Pearson’s correlation coefficient was calculated to identify the variables with a Pearson’s correlation coefficient of more than 0.60. Where two variables were highly correlated, the variable that was deemed more important based on expert judgment or theory was retained. Route length, stops, and population were correlated with average travel time, and so the former group of variables was removed. Route speed, jobs, number of households paying more than 30% for shelter, and density were all correlated with route accessibility, and so were removed. Monthly and single trip fare, minimum wage, gas price, and the percentage of buses available were all correlated with the dummy variable for COVID-19, and so these were removed. The two variables related to bicycle-sharing—total BIXI days and BIXI trips per day—were highly correlated with seasonal dummies, leading to their exclusion. To measure how the relationship between the independent variables and ridership evolved post-COVID-19, interaction variables were generated. This was done by multiplying each of the independent variables with the COVID-19 dummy variables (except for the seasonal dummy variables).

The data are organized in a longitudinal panel, with 12 repeated observations for each route—one for each period. Transport researchers have used different approaches to model transit ridership over time, including fixed-effect models (*56*, *57*), random-effect models (*14*), and linear mixed-effects models (also called linear mixed models) (*34*). A mixed-effects model contains both fixed and random effects (*58*, *59*). In this model, the fixed effects are represented by the independent variables’ coefficients. These represent how each independent variable is expected to affect the dependent variable as it increases or decreases. This model also has two random effects. Firstly, each route has a different random intercept: this represents the deviation from the model constant that is different for each route (i.e., between-route variation). The heterogeneity represented by the random intercept is unique to each route and assumed to be independent (i.e., uncorrelated) from the model’s independent variables. Secondly, each observation has a random residual: this represents the deviation from the model slope that is different for each observation (i.e., within-route variation).

Mixed-effects models with different explanatory variables were tested in Stata 16.1. The first model that was run included all the independent variables that were retained after the colinear variables were excluded. For four of these variables, both the variable and the variable interacted with the COVID-19 dummy were found to be statistically insignificant. Omitting these variables did not affect other coefficients’ weights, and so they were removed. These variables were route accessibility and the express route dummy. The variables interacting 10-Minute Max routes and Connects to Metro routes with COVID-19 were insignificant and were excluded. The two operations variables included in the first model—number of daily trips and average travel time—directly affect route accessibility, and a second model was estimated where route accessibility replaced these two operations variables. This enabled us to estimate the impact of accessibility on ridership separately. Besides removing the two operations variables and their interactions with the COVID-19 dummy, all the variables included in the first model were retained.

Sensitivity analyses were run for both models to understand how predicted daily ridership would change based on different trip frequencies and route accessibilities, respectively. For both sensitivity analyses, the means from October 2018 and October 2022 were inputted into all other variables. For the dummy variables relating to trip type (e.g., Connects to Metro, 10-Minute Max), the mode was inputted (e.g., more than 50% of routes connected to the metro, so that variable was inputted as 1). For the temporal dummy variables, the month was assumed to be October, and the year dictated whether COVID-19 was inputted as zero or one. Finally, a validation step was performed, in which the two models estimated were run on the testing data to validate their accuracy.

## Findings

### Summary Statistics

**Figure 3.**
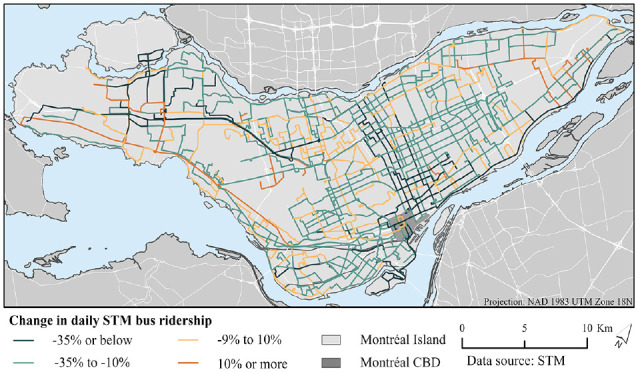
Change in daily Société de transport de Montréal (STM) bus ridership, by route, between October 2018 and October 2022. *Note*: CBD = central business district.

Route-level changes in ridership between October 2018 and October 2022 are shown in Figure 3. Decreases are most extreme near the CBD and the eastern center, while ridership is more resilient in the west and north. This map is very similar to changes in route frequency. Table 2 groups the routes by percentile, based on how their trip frequency increased or decreased between October 2018 and October 2022. The “Mean # of October 2018 trips” column indicates that reductions in service have been concentrated in high-frequency routes: the bottom percentiles had the highest mean number of trips before the pandemic. Almost 80% of routes historically identified as 10-Minute Max routes—theoretically the system’s most important—were in the bottom 25% percentiles. The last column demonstrates the high association between route reductions and ridership decline, with the routes suffering the greatest service reductions having the greatest ridership decline.

**Table 2.** Summary Statistics, with Routes Grouped by Change in Trip Frequency between October 2018 and October 2022

Percentile—change in trip frequency	Change in trip frequency from October 2018 to October 2022	Mean # of October 2018 trips	# of ex-10-Minute Max routes^b^	Change in ridership from October 2018 to October 2022
Top 5% routes with greatest increase in frequency	22%	64	0	31%
Top 10%	5%	56	0	−9%
Top 25%	2%	74	0	−9%
Rest of routes^a^	−4%	82	7	−7%
Bottom 25%	−18%	155	11	−16%
Bottom 10%	−24%	198	5	−23%
Bottom 5% with greatest decrease	−35%	196	7	−33%

a Routes from 25% to 75%.

b Route 139 was excluded from study because it overlaps with the new bus rapid transit.

### Model Results

Table 3 shows the results of the two mixed-effects models that were developed using the natural logarithm of daily route ridership as the dependent variable. The two models include the influence of transit operations on ridership in different ways: Model A via the inclusion of daily weekday trips and average weekday travel time, and Model B via including accessibility to jobs.

**Table 3.** Model Results

Variable name	A. Trips model			B. Accessibility model		
	Coef.	*z*	*P* > *z*	Coef.	*z*	*P* > *z*
Internal factors						
Daily trips (ln)	1.30	33.9	0.00			
Travel time (ln)	0.53	7.2	0.00			
Accessibility (ln)				0.11	4.9	0.00
10-Minute Max	0.09	4.4	0.00	0.09	3.9	0.00
Connects to metro	0.19	3.9	0.00	0.14	2.3	0.02
Connects to Exo	0.09	3.2	0.00	0.08	2.3	0.02
Intersects CBD	−0.15	−1.5	0.14	−0.18	−0.6	0.56
External factors						
Income (ln)	−0.11	−0.7	0.46	0.48	2.4	0.02
Recent immigrants (ln)	0.19	4.1	0.00	0.22	3.8	0.00
Unemployment rate (ln)	−0.51	−4.0	0.00	−0.61	−4.0	0.00
Work from home (ln)	−0.07	−0.9	0.37	−0.02	−0.2	0.87
Month of June	−0.18	−14.3	0.00	−0.23	−14.9	0.00
Month of August	−0.01	−0.8	0.43	0.01	0.3	0.73
Month of October	0.00	0.2	0.86	0.02	1.2	0.24
COVID-19	−1.17	−1.1	0.27	−3.28	−2.3	0.02
Interactions with COVID-19						
Daily trips (ln)	0.21	12.5	0.00			
Travel time (ln)	0.20	6.2	0.00			
Accessibility (ln)				0.04	2.98	0.00
Connects to Exo	−0.07	−3.8	0.00	−0.05	−2.11	0.04
Intersects CBD	−0.11	−3.3	0.00	−0.21	−5.42	0.00
Income (ln)	−0.14	−1.6	0.11	0.23	2.16	0.03
Recent immigrants (ln)	−0.11	−4.1	0.00	0.02	1.13	0.26
Unemployment rate (ln)	0.64	6.7	0.00	0.31	2.95	0.00
Work from home (ln)	−0.12	−2.5	0.01	−0.26	−4.95	0.00
Constant	−1.10	−0.7	0.51	0.85	0.36	0.72
Log-likelihood	496.77			141.86		
AIC	−943.54			−237.71		
BIC	−810.11			−114.96		
ICC	0.85			0.98		
Observations	1536			1536		
Number of groups	128			128		
Random-effects parameters	Estimate	95% CI		Estimate	95% CI	
SD of constant	0.35	0.30	0.41	1.15	1.01	1.31
SD of residual	0.15	0.14	0.15	0.00	0.16	0.18

*Note*: CBD = central business district; AIC = Akaike information criterion; BIC = Bayesian information criterion; ICC = intraclass correlation; SD = standard deviation; CI = confidence interval.

In Model A, daily weekday trips had a statistically significant positive relationship with ridership, with a coefficient of 1.30. This suggests that a 10% decrease in weekday trips leads to a 13% decrease in ridership, all else being equal. Daily weekday trips interacting with the COVID-19 dummy was statistically significant, with a coefficient of 0.21. This implies that at 10% decrease in trips post-COVID-19 will decrease ridership by 15.1%—13.0% + 2.1%. This suggests that demand is more elastic than previously, because ridership post-COVID-19 is more influenced by trip frequency. Average weekday travel time was positive and statistically significant both in its non-interacted form, and when interacted with the COVID-19 dummy. This suggests that routes with longer travel time, which typically cover greater distance, attract more riders by serving a great population. The 10-Minute Max dummy variable was statistically significant, indicating that riders responded positively to the marketing of certain routes as frequent, even when holding all other variables constant. When interacted with the COVID-19 dummy, it was insignificant—although this may be because the number of 10-Minute Max routes was materially reduced during COVID-19. Two variables demonstrate the decline in commuting trips into the CBD post-COVID-19. Whether a bus route intersected with the CBD was insignificant pre-COVID-19, but was significant when interacted with COVID-19. Connecting to a commuter rail station was positive and statistically significant in its un-interacted form, but negative and statistically significant when interacted. Pre-COVID-19, connecting to an Exo station led to a 9% increase in ridership, all else equal. The coefficient on the interacted Connects to Exo variable is −0.07. As such, connecting an Exo station post-COVID-19 only leads to a 2% increase in ridership (i.e., 9% − 7%). This is an intuitive result: the Exo system suffered from greatly reduced ridership after COVID-19, as it mostly served office workers commuting from the suburbs. It is reasonable that connecting to an Exo station would have a negligible effect post-COVID-19. Telework’s rising influence on travel patterns after COVID-19 was demonstrated by the WFH variable. The coefficient for WFH was always negative, but only became significant when interacted with the COVID-19 dummy variable.

In Model B, the two operations variables (trips and travel time) were replaced with accessibility to jobs. Accessibility had a positive and statistically significant relationship with ridership, albeit smaller. Model B suggests that a 10% increase in route accessibility would lead to a 1.1% increase in ridership, ceteris paribus. However, the importance of accessibility is largely unchanged since COVID-19—accessibility interacted with the COVID-19 dummy variable had a very small coefficient (0.04), indicating that increases in accessibility after COVID-19 would have largely the same effect as before. Several variables’ coefficients differed materially between models. In Model B, median household income was positively associated with ridership in both its interacted and non-interacted forms, while in Model A it was never significant. The relationship between ridership and the number of recent immigrants interacted with COVID-19 was surprisingly significant and negative in the trips model, but was insignificant in the accessibility model.

On top of these fixed effects, each model also has random effects. As described in the *Methodology* section, these consist of a random constant and residuals. For Model A, the standard deviation of this constant was 0.35 (meaning that the average route’s constant is ±0.35 points from the overall constant), while for Model B, this deviation was 1.15.

The intraclass correlation (ICC) is calculated by dividing the between-route variance from the total variance (between-route and within-route). It articulates what percentage of the error term is between-route variance (i.e., accounted for by the random intercept) and what is within-route variance (contained within the residuals). ICC scores can be used to assess the reliability of the statistical model, with a threshold of above 0.8 often used (*60*). Since Model A has an ICC score of 0.85 and that of Model B is 0.98, both models clear this threshold, showing high reliability. Model A had materially lower Akaike information criterion (AIC) scores (−943 compared to −237), suggesting it fits the data better.

### Sensitivity Analyses

**Figure 4.**
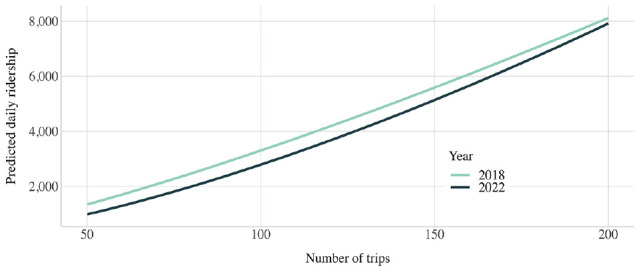
Sensitivity analysis of ridership and trips.

**Figure 5.**
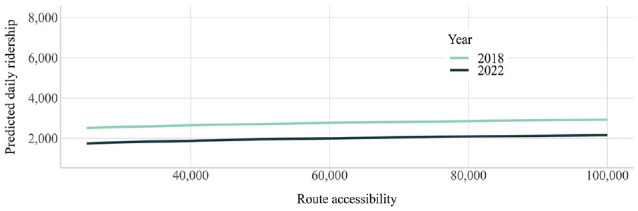
Sensitivity analysis of ridership and accessibility.

Sensitivity analyses were run for both models to understand how predicted daily ridership would change based on different trip frequencies and route accessibility. While the model was estimated in log–log form, the results were transformed into non-log form for plotting purposes. These two analyses highlight trip frequency and route accessibility’s different elasticities. Trip frequency is highly elastic—even more so after COVID-19—such that increasing trip frequency rapidly increases trip ridership (Figure 4). In contrast, accessibility is quite inelastic, suggesting that increases in accessibility will have a diminishing impact (Figure 5). In both cases, the intercept for 2022 is lower than that for 2018, suggesting that a certain level of trip frequency or accessibility in 2022 will achieve a lower ridership as compared to 2018.

### Model Validation

To cross-validate the models, both models were run using the testing data. The mean *predicted* log of ridership was 7.63 for the trips model, and 7.67 for the accessibility model (compared to an actual mean of 7.74). The Pearson’s correlation between the predicted and the actual ridership was calculated for both models. The trips model was far superior, registering a correlation of 0.95, compared to 0.53 for the accessibility model. The trips model performed better with respect to the root-mean-square-error (RMSE), achieving a value of 0.40 compared to 1.05 for the accessibility model.

## Discussion and Conclusion

This research examined the relationship between ridership and several operations and socioeconomic variables to identify the pre- and post-COVID-19 elasticities of transit demand. Many transit agencies are considering reducing service because of lower ridership, and so it is important to understand how the link between operations and ridership has evolved after the pandemic.

The study found that demand for transit was highly elastic and had grown more elastic since COVID-19. The rise of telework may partially explain this increase in elasticity. When faced with a service cut, workers who before COVID-19 would have been forced to endure a longer commute might now respond by commuting to the office less frequently. Rising car ownership, including among low-income households (*61*), and increases in active mode use for non-work purposes (*62*) may mean that individuals are more able to switch to a different mode if the transit route they use suffers a service reduction. This increase in elasticity has grave consequences for agencies, as it suggests the risk of a doom spiral is high. If transit agencies cut services, then they can expect to lose more riders, worsening their fiscal position. This suggests that further public funding for transit operations is necessary to stave off a total collapse of the system.

If this is not possible, then the findings suggest that agencies should attempt to maintain services on higher frequency routes and make reductions on less popular and low-frequency routes while accounting for equity issues. The study found that a 10% increase in route frequency led to a 15% increase in ridership. As the sensitivity analysis highlights, this exponential relationship means a reduction from 200 buses a day to 190 will lead to a greater decrease in ridership than going from 100 to 90. This might not be the case at extreme levels of frequency, because short headways can exacerbate bus bunching (*63*) and because shorter waits have diminishing benefits past a certain level. However, in the medium range, the relationship between frequency and ridership implies that riders are highly responsive to decreases in frequency post-COVID-19. This may be because even a small reduction in frequency for a high-frequency route can make a material difference in the customer experience (e.g., by forcing riders to check the schedule where previously they did not have to, or by subjecting them to overcrowding) (*64*). This finding is further supported by the 10-Minute Max variable being significant: all else being equal, routes marketed as 10-Minute Max had 9% more ridership. This implies that consumers are reacting positively to a branded bus service that promises a superior, more reliable customer experience (*21*). Our model implies that if cuts are necessary, agencies should focus on maintaining services on “core” and branded routes to maintain ridership levels as much as possible, while considering the equity implications of such a policy.

The study found that bus routes serving areas with more recent immigrants had higher ridership pre-COVID-19 and post-COVID-19. However, this “immigrant ridership boost” was lower post-COVID-19. This may suggest that immigrants were more likely to invest in alternative modes during the pandemic. It could also imply that immigrants continued to associate public transit with increased COVID-19 health risks (and as such were less likely to use it). Alternatively, it is possible that the *quality* of public transit service in immigrant neighborhoods declined disproportionately, and that this result is accounting for that decrease in quality. This finding suggests that agencies should investigate whether the customer experience for immigrant riders has uniquely changed, and remedy this as needed.

The study found a small relationship between accessibility to jobs and ridership. This is a surprising finding, given the significant literature demonstrating the relationship between accessibility and transit mode share. This may be because accessibility is traditionally calculated as a system-wide measure, rather than a route-level measure. Further research is required to investigate whether accessibility has declined in importance since COVID-19.

Future research may examine how the elasticity of demand differs between different types of transit (e.g., between buses and heavy rail) and between different subgroups (e.g., between students and workers). However, even if certain riders *are* more inelastic (e.g., captive riders), agencies should be mindful in making cuts disproportionately in those areas; socially regressive cuts would undermine transit’s equity objectives.

Future studies could also include additional variables that might affect transit demand. This includes the impact of service quality, including reliability (i.e., on-time performance), which may have become a more important factor since COVID-19. Studies could also investigate the significance of changing rates of car ownership on transit demand, which was not available for our level of analysis. Research could also use multiple dummy variables to characterize the post-COVID-19 time period (rather than just one) to account for increased pressure over time on employees to return to in-person work. This could shed light on the impact on transit ridership of certain employers instituting firmer return-to-office mandates.
